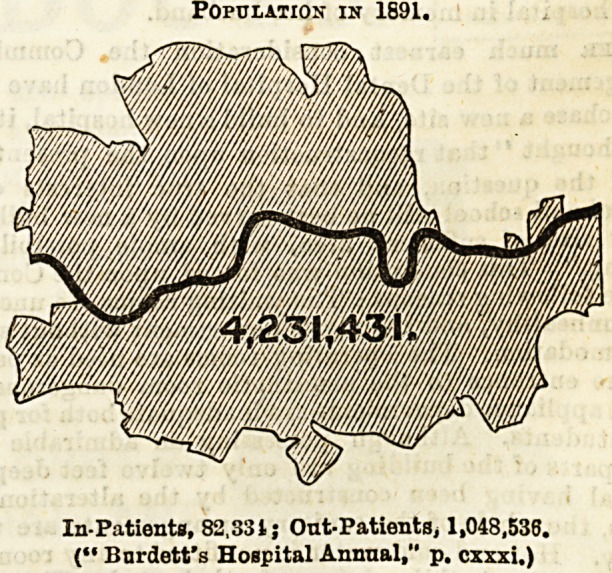# The Poor of London and Their Medical Needs

**Published:** 1893-03-25

**Authors:** Conrad W. Thies

**Affiliations:** Secretary Royal Free Hospital, London


### THE POOR OF LONDON AND THEIR MEDICAL NEEDS.

By Mr. CONRAD W. THIES, Secretary Royal Free Hospital, London.

The subject which I venture to bring before this meeting of the Hospitals Association certainly cannot claim the merit of novelty, indeed, it is "a tale oft told," and yet it is one of perennial interest, demanding the earnest attention of all who are in any way engaged in the work of providing for the medical necessities of the poorer classes of the metropolis.

The special object which I have had in view in the preparation of this paper has been to lay before you some facts respecting the economic condition of the metropolitan population, in order thereby to show what are the actual numbers of the poorer classes, on whose behalf it may be fairly assumed that, owing to their poverty, they are fit and proper recipients of the benefits of charitable medical relief.

I am fully persuaded that these facts, if carefully considered, will tend to remove the impression, which is evidently very commonly entertained, that the provision made for free medical relief in London is already excessive. This opinion was strongly expressed by several well qualified witnesses before the Lords' Committee on Metropolitan Hospitals, and occasional articles in the public press have severely criticised the voluntary medical charities, on the assumption of the truth of this view of the question. This is no new criticism, however, for I find the same view was held by a very competent authority—Dr. John Chapman—who recorded his opinion over twenty years ago as follows: "The enormous proportion of the population of London who are the recipients of medical charitable relief cannot fail to strike with astonishment anyone who considers it for the first time. Indeed, it seems at first sight incredible that, in the wealthiest metropolis in the world, medical charity should have assumed the colossal magnitude which it actually presents."

There never was a period in our history when more universal interest was manifested in the condition of our poorer neighbours than during the past few years. This general awakening of the public conscience to its social responsibilities has been the result of a combination of causes to which I need not now refer, and it has been shown in various ways. We have had elaborate reports by Royal Commissions, by philanthropic and religious societies, and on every hand we find that efforts are being made to brighten the lives of the poor, and to ameliorate the distress and suffering which is inseparable from extreme poverty.

One effect of this revival of interest in social questions has been the opening up of fresh channels for the public benevolence, and there is a danger lest the medical charities should be neglected, or, at least, not receive their due share of public support. I find, for instance, that in the last edition of Howe's "Directory of the Metropolitan Charities" it is estimated that the total income of 980 charitable institutions in London for the year 1891 was £6,246,136, of which sum about one-half was absorbed by missionary societies, while the voluntary medical charities received about £630,000. This state of affairs has certainly not resulted from any lack of enterprise on the part of the medical charities, for the public generally, and philanthropic persons in particular, are persistently deluged with appeals on their behalf, until, by their very very frequency, even the most importunate and smartly-written presentment of the case for the hospitals meets with comparatively little response.

We had a notable illustration of this fact during the past year, when "Suffering London" was published, and special efforts were put forth to stimulate the public interest in our medical charities. I thought at the time that probably a statement of the simple facts would have proved more effective in enlisting the sympathies and opening the purse strings of the well-to-do classes. I have since endeavoured to ascertain what is really known as to the actual conditions under which the poor live, and then, furnished with this information, to consider whether the provision made for their medical needs is really excessive.

Before referring to the result of my inquiries, I would ask your attention to a few facts respecting London. Very few persons, even of those who have for many years resided in the metropolis, realise what London really means, or the magnitude of the problems arising out of the enormous aggregation of people which make up the metropolitan population. It must be borne in mind that the area covered by the Greater London is much larger than that comprised in the County of London, for on every side of the county limits there stretches a belt of suburbs and towns, extending as far as Barnet on the north, and Croydon on the south, which, practically, for the purposes of hospital relief, are a part of London. The population of this enlarged area is now nearly six millions, that is considerably more than that of the whole of Ireland. If it were emptied to-morrow the whole of the inhabitants of Scotland and Wales together could barely refill it, and the three next largest cities in the world could be combined without outnumbering its millions.

Its growth has gone on at a prodigious rate since the beginning of this century, and notably so during the past twenty years. A mere statement of the figures conveys but a faint impression of its magnitude, but I am enabled to reproduce two maps of the county of London, which were

POPULATION IN 1871.

In-Patients, 58,671; Out-Patients, 830,519.
(Dr. John Chapman, "Medical Charity," p. 6.)

POPULATION IN 1891.

In-Patients, 82,331; Out-Patients, 1,048,536.

("Burdett's Hospital Annual," p. cxxxi.)

published in the Hospital Sunday Supplement of the *Lancet* in June last. These maps show at a glance the enormous growth of the population during the past twenty years. The mere addition to the population during that period is equal to the entire population of three large cities, such as Manchester, Leeds, and Norwich added together.

It will be seen from the above figures that with the enormous growth of the population there has been a proportionate increase in the number of both in and out patients treated by the medical charities; and it is not very surprising, therefore, to find that the opinion expressed by Dr. John Chapman in 1871 is still very commonly entertained in the present day.

Previously to the publication of Mr. Charles Booth's remarkable work, "The Labour and Life of the People of London," the condition of this mass of population was a matter of conjecture, based upon merely local inquiries and partial knowledge. To quote Mr. Booth's own words: "The real London was hidden behind a curtain, on which were painted terrible pictures of starving children, suffering women, overworked men, horrors of drunkenness and vice, monsters and demons of inhumanity, giants of disease and despair. Did these pictures truly represent what lay behind, or did they bear to the facts a relation similar to that which the pictures outside a booth at some country fair bear to the performance or show within?" This curtain has now been lifted by Mr. Booth and his co-workers, after five years steady work, and we are enabled thereby to see clearly the conditions under which the poorer classes of the people live: the revelation exposes a state of things which is very startling, and certainly not creditable to our modern civilisation. It is evident that the saying, "The poor always ye have with you," which has been applicable to all ages and countries in the past, is especially true as applied to the London of to-day. Enthusiastic social reformers may confidently predict a future for humanity when poverty and destitution will be unknown, and where the material wants of every member of the community will be assured, but unfortunately there are no prospects of "the good time coming" being realised in our day.

## Figures and Tables

**Figure f1:**
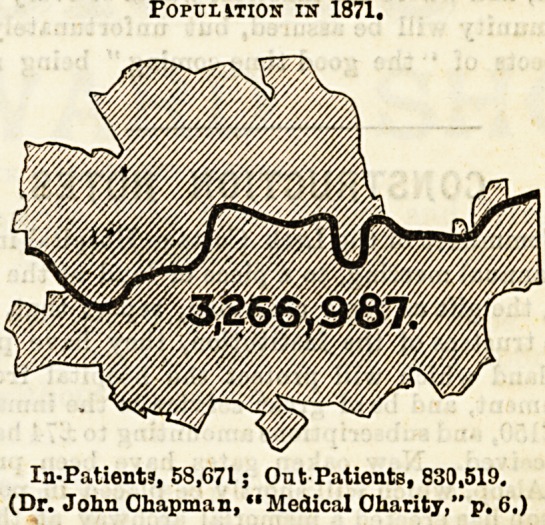


**Figure f2:**